# Similar HIV protection from four weeks of zidovudine versus nevirapine prophylaxis among formula-fed infants in Botswana

**DOI:** 10.4102/sajhivmed.v19i1.751

**Published:** 2018-03-28

**Authors:** Kathleen M. Powis, Shahin Lockman, Gbolahan Ajibola, Michael D. Hughes, Kara Bennett, Jean Leidner, Oganne Batlang, Kerapetse Botebele, Sikhulile Moyo, Erik van Widenfelt, Joseph Makhema, Chipo Petlo, Haruna B. Jibril, Kenneth McIntosh, Max Essex, Roger L. Shapiro

**Affiliations:** 1 Massachusetts General Hospital, Departments of Medicine and Pediatrics, United States; 2 Harvard T. H. Chan School of Public Health, Department of Immunology and Infectious Diseases, United States; 3 Botswana Harvard AIDS Institute Partnership, Gaborone, Botswana; 4 Brigham and Women’s Hospital, Infectious Disease Division, United States; 5 Bennett Statistical Consulting, Inc, Ballston Lake, United States; 6 Goodtables Data Consulting, Norman, United States; 7 Ministry of Health, Gaborone, Botswana; 8 Division of Infectious Diseases, Boston Children’s Hospital, United States

## Abstract

**Background:**

The World Health Organization HIV guidelines recommend either infant zidovudine (ZDV) or nevirapine (NVP) prophylaxis for the prevention of intrapartum mother-to-child HIV transmission (MTCT) among formula-fed infants. No study has evaluated the comparative efficacy of infant prophylaxis with twice daily ZDV versus once daily NVP in exclusively formula-fed HIV-exposed infants.

**Methods:**

Using data from the Mpepu Study, a Botswana-based clinical trial investigating whether prophylactic co-trimoxazole could improve infant survival, retrospective analyses of MTCT events and Division of AIDS (DAIDS) Grade 3 or Grade 4 occurrences of anaemia or neutropenia were performed among infants born full-term (≥ 37 weeks gestation), with a birth weight ≥ 2500 g and who were formula-fed from birth. ZDV infant prophylaxis was used from Mpepu Study inception. A protocol modification mid-way through the study led to the subsequent use of NVP infant prophylaxis.

**Results:**

Among infants qualifying for this secondary retrospective analysis, a total of 695 (52%) infants received ZDV, while 646 (48%) received NVP from birth for at least 25 days but no more than 35 days. Confirmed intrapartum HIV infection occurred in two (0.29%) ZDV recipients and three (0.46%) NVP recipients (*p* = 0.68). Anaemia occurred in 19 (2.7%) ZDV versus 12 (1.9%) NVP (*p* = 0.36) recipients. Neutropenia occurred in 28 (4.0%) ZDV versus 21 (3.3%) NVP recipients (*p* = 0.47).

**Conclusions:**

Both ZDV and NVP resulted in low intrapartum transmission rates and no significant differences in severe infant haematologic toxicity (DAIDS Grade 3 or Grade 4) among formula-fed full-term infants with a birthweight ≥ 2500 g.

## Introduction

Previous studies have demonstrated that infant antiretroviral (ARV) prophylaxis with zidovudine (ZDV) or nevirapine (NVP), or both combined, have been efficacious in preventing intrapartum mother-to-child HIV transmission (PMTCT).^1,2,3,4,5^ Recent work has shown that infant NVP prophylaxis provides additional protection against mother-to-child HIV transmission (MTCT) during breastfeeding.^4,5^ Currently, the World Health Organization (WHO) recommends NVP prophylaxis for all HIV-exposed infants, but WHO guidelines do not preferentially support either ZDV or NVP in formula-fed HIV-exposed infants.^6,7^ In fact, the strength of the recommendation for use of daily NVP or twice daily ZDV from birth for 4–6 weeks among breastfed HIV-exposed infants was categorised as ‘strong’ based on ‘moderate’ evidence.^6^ However, the WHO prophylaxis recommendation for formula-fed HIV-exposed infants from birth was categorised as a ‘conditional’ recommendation with ‘low’ evidence, suggesting the need for data-driven evidence.^6^

Formula feeding is an alternative to breastfeeding in lower infant-mortality settings where formula feeding is available and can be safely prepared; when breastfeeding cannot occur; and among those who wish to maximally reduce the risk of late HIV transmission. At present, formula feeding is widely practiced as a PMTCT strategy throughout Europe and the Americas, and in some areas of Asia and Africa.^8,9,10,11^

The Mpepu Study, conducted in Botswana from May 2011 through June 2015, permitted comparison of four weeks of infant prophylaxis consisting of either single-dose NVP (sdNVP) and ZDV twice daily or NVP once daily among formula-fed HIV-exposed infants. The majority of infants born to women who consented prior to February 2013 received sdNVP and ZDV prophylaxis. Thereafter, most infants received NVP prophylaxis as a single-drug regimen. We performed a retrospective analysis evaluating MTCT rates and haematologic safety of ARV prophylaxis for full-term, normal birthweight, formula-fed infants who received 4 weeks of sdNVP plus ZDV versus NVP.

## Study population and methods

### Study population and monitoring

This retrospective study was conducted in Botswana, a middle-income country with high HIV prevalence, estimated at 21.9% among persons aged 15–49 as of 2016.^12^ Botswana was among the first countries with high HIV prevalence to promote PMTCT, offering free antiretroviral treatment (ART) to pregnant women living with HIV who qualified for treatment for their own health as early as 2002, while providing ZDV monotherapy to pregnant women living with HIV if they did not yet qualify for HIV treatment. In 2012, Botswana national HIV treatment guidelines were updated to recommend triple ARV use among all pregnant women living with HIV, regardless of their HIV disease state.^13^ As of 2016, more than 95% of pregnant women living with HIV in Botswana accessed ART.^14^ This has contributed to significant reductions in MTCT with a rate of 1.8%, despite the fact that HIV prevalence among pregnant women remains high at over 25%.^15^ Up until 2016, Botswana has promoted exclusive formula feeding in the first six months-of-life for HIV-exposed infants.^13,16^ In 2016, this policy changed to encourage breastfeeding of HIV-exposed uninfected (HEU) infants so long as the mother is taking ART and is virally suppressed.^17^ Despite this policy change, the practice of providing free infant formula-fed during the first year of life to HIV-exposed infants remains an option. Use of formula feeding for HIV-exposed infants has been widely adopted, with nearly 80% of women living with HIV in Botswana opting to formula feed their infant. In this context, data were analysed retrospectively from the Mpepu Study to evaluate comparative efficacy of four weeks of sdNVP with ZDV versus NVP.

The Mpepu Study, a double-blinded randomised controlled trial, investigated prophylactic co-trimoxazole (CTX) versus placebo from 2 weeks to 15 months of age in HEU infants, studying health outcomes including diarrhoeal illness, pneumonia and death (NCT01229761). This study has previously been described.^18^ In brief, HIV-infected women were eligible for enrolment between 26 weeks gestation and 35 days postpartum. Infants were enrolled from birth to 35 days-of-life. The study opened for enrolment in May 2011 and was conducted in the capital city (Gaborone), the rural village of Molepolole, located approximately 56 km from the capital city, and the peri-urban town of Lobatse located approximately 67 km from the capital city. Consistent with Botswana’s PMTCT guidelines,^19^ the initial study protocol followed government dosing of sdNVP (6 mg) within 72 h of birth and ZDV [4 mg/kg twice daily for full-term infants (37 weeks) with a birth weight ≥ 2500 g] for four weeks. Randomisation to CTX or placebo took place between 28 and 35 days-of-life. From February 2013, the protocol was amended to allow randomisation to CTX or placebo as early as 14 days-of-life for infants born full-term and weighing ≥ 2500 g. To avoid the possibility of overlapping haematologic toxicity from CTX with ZDV with this protocol change, study infants were offered NVP (15 mg once daily for four weeks from birth). Of note, the prevailing prophylaxis under national guidelines remained sdNVP with four weeks of ZDV.^20^ Therefore, some infants enrolled in the study after the protocol amendment received sdNVP and ZDV initiated by government nursing staff at maternity wards where they were born.

Data were collected on the initiation and stop dates of infant ARV prophylaxis. At infant enrolment and randomisation study visits, infant HIV-1 testing was performed using qualitative polymerase chain reaction (PCR) DNA assay (Amplicor HIV-1, Roche Diagnostic Systems, New Jersey, USA) for infants at the birth (enrolment) and randomisation study visits. Over 95% of infant enrolment DNA PCRs were collected in the first 72 h after infant birth. If an infant enrolment PCR was not available and a subsequent HIV-1 PCR test was negative, the enrolment PCR was imputed as negative. Additional infant HIV DNA PCR testing was performed at six weeks of life in government-run health facilities in accordance with Botswana national PMTCT guidelines with results documented in Mpepu Study records and by Mpepu Study clinicians at any visit where an infant was noted to have significant interim or current illness or insufficient weight gain. Infants attending the 18-month study visit were retested for HIV status using enzyme-linked immunosorbent assay (ELISA). A full blood count was drawn at randomisation (between 14 and 35 days-of-life), and 3- and 6-month visits and included haemoglobin and absolute neutrophil count. Infant haemoglobin and absolute neutrophil count (ANC) values were graded using the Division of AIDS (DAIDS) Table for Grading and Severity of Adult and Paediatric Events, Version 1.0, December 2004; Clarification August 2009.^21^ Haemoglobin or absolute neutrophil count results corresponding to DAIDS Grade 3 or Grade 4 values were classified as anaemia or neutropenia.

Maternal verbal reports and clinical records were used to ascertain maternal ARV use; all maternal ARVs were provided through the government’s Infectious Disease Care Clinics. At Mpepu Study inception, the national policy endorsed triple ARV for all HIV-infected women for PMTCT (Option B),^19^ transitioning from a policy where only women with CD4+ cell counts of < 350 cells/µL were eligible for triple ARVs.^20^ However, Option B was not fully operationalised until January 2013.^22^

### Study inclusion and exclusion criteria

Infants were eligible for this secondary analysis if they were born full-term (≥ 37 weeks gestation) with a birth weight ≥ 2500 g, received 25–35 days of ZDV twice daily or NVP once daily and were exclusively formula-fed in the first 35 days-of-life based on maternal verbal report. Where mixed feeding was reported, infants were excluded. National guidelines endorsed sdNVP within 72 h of birth for all infants receiving ZDV prophylaxis. Gestational age and birth weight restrictions were employed to avoid study-specific sources of bias in this secondary analysis, because preterm and low-birth-weight infants in the later study period were more likely to have received ZDV prophylaxis.

### Statistical methods

SAS, version 9.3 (SAS Institute, Cary, North Carolina, USA) was used for statistical analyses. Maternal and infant characteristics were compared by infant ARV prophylaxis group. Continuous variables were compared using Wilcoxon rank sum test. Fisher’s exact testing was used for comparison of non-continuous variables and to assess MTCT prevalence by infant HIV DNA PCR results obtained between 14 and 35 days-of-life. Exact (Clopper–Pearson) methods for binomial proportions were used to estimate the MTCT confidence limits. Time to first occurrence of infant anaemia or neutropenia in the first six months-of-life by ARV prophylaxis group was compared using Cox proportional hazard models, stratified by infant randomisation arm (CTX or placebo). All testing used a significance level of 0.05, with two-sided hypothesis testing.

#### Ethical consideration

The Health Research Development Committee of Botswana and the Office of Human Research Administration at the Harvard T.H. Chan School of Public Health approved the study protocol and amendments. Women signed a written consent form approved by the ethical review boards for their and their infants’ participation.

**Project research number:** Clinical Trials.gov Registration Number: NCT01229761.

## Results

**FIGURE 1:**
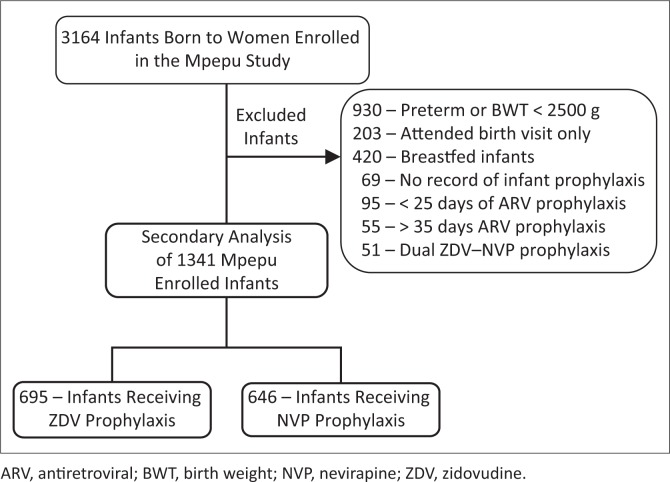
Consort diagram.

Of 3164 infants enrolled in the Mpepu Study, 1823 (58%) infants were excluded from this secondary analysis: 930 born preterm and/or with a birth weight < 2500 g, 203 evaluated only at birth, 420 breastfed infants, 69 without ARV prophylaxis documentation, 95 with documentation of < 25 days of prophylaxis, 55 with extended prophylaxis (> 35 days) and 51 given dual ZDV and NVP prophylaxis (Figure 1). Of the remaining 1341 (42%) infants, 695 received ZDV prophylaxis for a median of 28 [interquartile range (IQR) 27–30] days, with 665 (95.7%) of the infants receiving ZDV prophylaxis also having documentation of sdNVP dosing within 72 h of delivery. The remaining 646 infants received NVP for a median of 29 days (IQR 28–30). Before the protocol change in February 2013, 2 (0.3%) infants received NVP prophylaxis, whereas 644 (86.6%) infants received NVP prophylaxis after the change.

Characteristics of mother–infant pairs by prophylaxis regimen are presented in Table 1. A significantly higher proportion of mothers of NVP recipients received triple ARVs during pregnancy, as 99% of all NVP recipients were born after Botswana’s National HIV Treatment Guidelines transitioned to Option B. Only 1.3% of mothers of NVP recipients received ZDV monotherapy in pregnancy versus 21.4% of mothers of ZDV recipients. The median days to HIV DNA PCR testing at parent study randomisation (visit for initiation of CTX/placebo) was 29 days-of-life for ZDV infants and 15 days-of-life for NVP infants, reflecting the earlier age at randomisation for NVP recipients after the protocol change. Early closure of the Lobatse site led to a higher proportion of Lobatse enrolled infants receiving ZDV. In all other respects, characteristics between groups were similar.

**TABLE 1:** Comparison of mother–infant characteristic by infant antiretroviral prophylaxis.

Maternal or infant characteristics	Mothers† of infants with ZDV prophylaxis‡ (*n* = 693)	Mothers† of infants with NVP prophylaxis (*n* = 642)
Median maternal age (years) [IQR]	30.9 [ 26.9–34.8]	31.6 [ 26.8–36.0]
**Gravida including current pregnancy (*n*, %)**		
1	88 (12.7%)	93 (14.5%)
2	172 (24.8%)	136 (21.2%)
3	187 (27.0%)	171 (26.6%)
4 or more	246 (35.5%)	242 (37.7%)
Median enrollment CD4+ count (cells/µL) [IQR]	476 [348–629]	508 [352–674]
Enrollment CD4+ < 200 cells/µL (*n*, %)	35 (5.0%)	35 (5.6%)
**Maternal ARV regimen**		
Triple ARVs initiated before conception	247 (35.6%)	313 (48.8%)
Triple ARVs initiated in pregnancy	269 (38.8%)	299 (46.6%)
ZDV monotherapy	148 (21.4%)	8 (1.2%)
No ARVs	29 (4.2%)	22 (3.4%)
**Enrollment site (*n*, %)**§		
Molepolole (Village)	238 (34.3%)	246 (38.3%)
Lobatse (Town)	104 (15.0%)	2 (0.3%)
Gaborone (City)	351 (50.7%)	394 (61.4%)
**Marital status (*n*, % )**		
Single	548 (79.2%)	533 (83.0%)
Married/Cohabitating	134 (19.4%)	105 (16.4%)
Widowed/Divorced	10 (1.4%)	4 (0.6%)
**Education (*n,* %)**		
None or Primary	117 (18.3%)	91 (15.7%)
Secondary	388 (60.7%)	370 (63.9%)
University	134 (21.0%)	118 (20.4%)
Infant characteristics	Infants with ZDV prophylaxis (*n* = 695)	Infants with NVP prophylaxis (*N* = 646)
**Infant sex (*n*, %)**		
Male	354 (50.9%)	322 (49.8%)
Female	341 (49.1%)	324 (50.2%)
Median gestational age in weeks at delivery [IQR]	39 [38–40]	39 [38–40]
**Median anthropometric measures**		
**Birth weight (kg) [IQR]**		
Male infants	3.12 [2.87–3.40]	3.10 [2.85–3.36]
Female infants	3.00 [2.80–3.25]	2.98 [2.78–3.20]
**Length (cm) [IQR]**		
Male infants	51 [49–53]	51 [49–52]
Female infants	50 [49–52]	50 [49–51]
Median days of ZDV or NVP prophylaxis [IQR]	28 [27–30]	29 [28–30]
**Mpepu Study randomisation arm (n, %)**		
Co-trimoxazole	359 (51.7%)	309 (47.8%)
Placebo	336 (48.3%)	337 (52.2%)
Median days to HIV DNA PCR testing [IQR]	29 [28–30]	15 [14–16]

ARVs, antiretrovirals; IQR, interquartile range; NVP, nevirapine; ZDV, zidovudine; cm, centimeters; kg, kilograms.

† , Of the 1335 women in this sub-study, 6 women delivered twins of which both infants met study eligibility criteria.

‡ , Among infants receiving ZDV prophylaxis, 95.7% had documentation of single-dose NVP receipt within 72 h of delivery.

§ , The Lobatse study site closed to accrual in August 2012, prior to study protocol change to use NVP infant prophylaxis.

Of the 1341 formula-fed infants, 9 (0.67%) represented potential intrapartum infections (HIV infection acquired during labour or delivery), having a first positive HIV DNA PCR result > 72 h after delivery; 6 [0.86%; 95% confidence interval (CI): 0.32% – 1.87%] ZDV recipients and 3 (0.46%; 95% CI: 0.10% – 1.35%) NVP recipients (*p* = 0.51). Among these nine potential intrapartum infections, five were confirmed intrapartum infections, having documentation of an initial negative HIV DNA PCR prior to a positive test result on a second HIV DNA PCR test, two (0.29%; 95% CI: 0.04% – 1.04%) infants receiving ZDV and three (0.46%; 95% CI: 0.04% – 1.12%) receiving NVP (*p* = 0.68). The remaining four infants, all receiving ZDV prophylaxis (one without sdNVP dosing documentation), had their first HIV DNA PCR test between 6 and 29 days-of-life, and it was positive. For these infants, the possibility of *in utero* transmission cannot be excluded.

A total of 201 (15%) of the 1341 mother–infant pairs were at higher risk for vertical transmission, defined as no or < 28 days of maternal ARV use during pregnancy, or maternal enrolment CD4+ cell count < 250 cells/µL. Of these, 110 infants received ZDV and 91 received NVP. Four (2.0%) potential intrapartum transmission events occurred among high-risk infants; three (2.73%; 95% CI: 0.57% – 7.76%) ZDV recipients and one (1.10%; 95% CI: 0.03% – 5.97%) NVP recipient (*p* = 0.63). Two of the high-risk ZDV recipients (one without sdNVP dosing documentation) were not confirmed intrapartum transmissions, as their first HIV DNA PCR test was positive but was not drawn until more than two weeks after delivery.

A total of 31 (2.3%) infants experienced at least one episode of anaemia, by DAIDS Grade 3 or Grade 4 criteria, in the first six months: 19 (2.7%) ZDV recipients and 12 (1.9%) NVP recipients (*p* = 0.36). Because the parent study involved infant randomisation to CTX or placebo, and CTX can cause bone marrow suppression,^23^ Cox proportional hazard models were used to evaluate time to first occurrence of anaemia or neutropenia by infant prophylaxis with stratification by infant randomisation arm (CTX or placebo). The adjusted hazard ratio for the time to first episode of anaemia among ZDV recipients did not differ significantly from NVP recipients [aHR 1.51 (95% CI: 0.73–3.14), *p* = 0.26] after stratification. DAIDS Grade 3 or Grade 4 neutropenia occurred more frequently than anaemia, with 49 (3.7%) infants experiencing at least one episode in the first six months; 28 (4.0%) ZDV recipients and 21 (3.3%) NVP recipients (*p* = 0.47). Time to first episode of neutropenia did not differ significantly for ZDV recipients compared with NVP recipients [aHR 1.04 (95% CI 0.57–1.91), *p* = 0.90] after stratification by CTX versus placebo.

## Discussion

Using data from the Mpepu Study, we provide the first comparative evaluation of vertical transmission and haematologic safety among full-term, normal birthweight, formula-fed HIV-exposed infants, analysing infant prophylactic regimens of sdNVP and ZDV twice daily versus NVP once daily. Whether including confirmed or potential intrapartum transmissions, the intrapartum MTCT incidence was low in this cohort where over 80% of all women received triple ARVs during pregnancy. Reassuringly, vertical transmission was low for both infant prophylaxis regimens. Even among mother–infant pairs at highest risk for MTCT, both infant prophylaxis regimens achieved low vertical transmission outcomes. DAIDS Grade 3 and Grade 4 anaemia and neutropenia events were each noted in fewer than 5% of infants, with no significant difference between prophylaxis groups in the first six months-of-life.

This study reduced bias by reporting confirmed and potential intrapartum transmissions and utilising very similar comparison groups from each study era. However, the study design imposed some limitations to this secondary analysis. First, preterm and low-birth-weight infants in the Mpepu Study were more likely to receive ZDV. Unfortunately, this precluded analysis of transmission outcomes and haematologic safety among infants born at < 37 weeks gestational age or with a birth weight < 2500 g, excluding nearly 30% of the infants in the Mpepu Study. Second, it is possible that some false-negative HIV DNA PCR results may have occurred, because testing was performed at the time the infant was taking prophylaxis or within days of discontinuing prophylaxis. However, in a study conducted by Burgard and colleagues evaluating the sensitivity of HIV DNA PCR performed between 15 and 45 days-of-life among 1293 formula-fed HIV-exposed infants, the sensitivity of HIV DNA PCR during this window when infants were taking or had recently completed ARV prophylaxis was quite high (88%).^24^ Mpepu Study infants were referred for HIV DNA PCR testing at six weeks of age under Botswana’s national programme, with no positive tests known to the study team. Furthermore, 48% of formula-fed children had an 18-month ELISA through the study (the remaining 52% were not followed to 18 months owing to early closure of the study for futility, at the recommendation of the Data Safety and Monitoring Board); all but one of these children had a negative ELISA at 18 months-of-life (the one infant with a positive ELISA had documentation of a negative HIV DNA PCR at three months-of-life, suggesting HIV acquisition from a source other than intrapartum HIV transmission, such as undisclosed breastfeeding). Third, this analysis involved comparison of infants from two consecutive time periods. As such, temporal confounders may be present. For example, less extensive maternal ART coverage in the earlier study era may explain the non-significant increase in overall (but not confirmed) intrapartum transmission events with ZDV (6 vs. 3). In terms of haematological toxicity, we acknowledge that the Mpepu testing schedule with a full blood count obtained between 14 and 35 days-of-life with follow-up at 3- and 6 months-of-life may have missed the occurrence of anaemia or neutropenia shortly after the infant discontinued ARV prophylaxis but before the 3-month visit. Lastly, this is a retrospective analysis of a study specifically designed for other purposes. As such, it was not powered to detect prophylaxis efficacy between the two infant regimens. Although a larger study with more complete birth PCR testing might have provided better discrimination between NVP and ZDV, the very low overall occurrence of MTCT in the Mpepu Study suggests that clinically meaningful differences in transmission were not missed.

In conclusion, while formula feeding of HIV-exposed infants in resource-limited settings remains the exception, our study findings provide reassuring evidence that a 4-week infant prophylaxis regimen of either sdNVP plus ZDV or ongoing NVP was similarly efficacious for PMTCT among formula-fed, full-term, normal birthweight infants in the context of extensive maternal ART use, without significant differences in haematologic adverse consequences. Prior to this study, the WHO recommendation for the use of sdNVP plus ZDV versus NVP was categorised as ‘conditional’ and based upon low evidence. Our comparative analysis strengthens the evidence, supporting current WHO recommendations for use of either ZDV or NVP as prophylaxis among HIV-exposed full-term infants in the first month of life to prevent intrapartum HIV acquisition.
